# A phase 2 basket trial of combination therapy with trastuzumab and pertuzumab in patients with solid cancers harboring human epidermal growth factor receptor 2 amplification (JUPITER trial)

**DOI:** 10.1097/MD.0000000000021457

**Published:** 2020-08-07

**Authors:** Kenta Takahashi, Eri Ishibashi, Toshio Kubo, Yohei Harada, Hideyuki Hayashi, Masayuki Kano, Yasushi Shimizu, Hidekazu Shirota, Yukiko Mori, Manabu Muto, Chikashi Ishioka, Hirotoshi Dosaka-Akita, Hisahiro Matsubara, Hiroshi Nishihara, Naoko Sueoka-Aragane, Shinichi Toyooka, Akihiro Hirakawa, Ukihide Tateishi, Satoshi Miyake, Sadakatsu Ikeda

**Affiliations:** a Center for Innovative Cancer Treatment; b Medical Innovation Promotion Center, Tokyo Medical and Dental University, Tokyo; c Department of General Thoracic Surgery and Breast and Endocrinological Surgery, Okayama University Graduate School of Medicine, Dentistry, and Pharmaceutical Science, Okayama; d Division of Hematology, Respiratory Medicine and Oncology, Department of Internal Medicine, Faculty of Medicine, Saga University, Saga; e Genomics Unit, Keio Cancer Center, Keio University School of Medicine, Tokyo; f Department of Frontier Surgery, Chiba University, Chiba; g Department of Medical Oncology, Faculty of Medicine & Graduate School of Medicine, Hokkaido University, Hokkaido; h Department of Clinical Oncology, Tohoku University Hospital, Sendai; i Department of Therapeutic Oncology, Graduate School of Medicine, Kyoto University, Kyoto; j Clinical Research Center; k Department of Diagnostic Radiology, Tokyo Medical and Dental University, Tokyo, Japan; l Moores Cancer Center, University of California, San Diego, CA.

**Keywords:** basket trial, human epidermal growth factor receptor 2 amplification, pertuzumab, phase 2 trial, solid cancers, trastuzumab

## Abstract

**Introduction:**

Human epidermal growth factor receptor 2 (*HER2*) gene amplification and mutations have emerged as oncogenic drivers and therapeutic targets not limited to breast and gastric cancers, but also in a variety of cancers. However, even if an actionable gene alteration is found, the incidence of *HER2* amplification in these cancers is less than 5%. It is too difficult to conduct a conventional randomized, controlled trial in a rare fraction. Therefore, we have designed a organ-agnostic basket study, which covers a variety of solid cancers harboring *HER2* amplification, in 1 study protocol.

**Methods/Design:**

This trial is a multicenter, single-arm, basket phase 2 study in Japan. Patients with solid cancers harboring *HER2* amplification that have progressed with standard treatment, or rare cancers for which there is no standard treatment, will be eligible. Target cancers include bile duct, urothelial, uterine, ovarian, and other solid cancers where *HER2* amplification is detected by comprehensive genomic profiling using next-generation sequencing technology. A total of 38 patients will be treated with combination therapy with trastuzumab and pertuzumab every 3 weeks until disease progression, unmanageable toxicity, death, or patient refusal. The primary endpoint is the objective response rate, and secondary endpoints are progression-free survival, overall survival, and duration of response.

**Discussion:**

The aim of this trial is to evaluate the safety and efficacy of combination therapy with trastuzumab and pertuzumab in patients with locally advanced or metastatic, solid cancers harboring *HER2* amplification. Instead of focusing on 1 organ type, our trial design uses a basket study focusing on *HER2* amplification, regardless of the site or origin of the cancer. The results of our study will advance clinical and scientific knowledge concerning the treatment of locally advanced, rare solid cancers harboring *HER2* amplification, using the combination of trastuzumab and pertuzumab.

**Trial registration:**

This trial was registered in Japan Registry of Clinical Trials (jCRT) on February 25, 2019, as jRCT2031180150.

## Abstract 2

Supplemental Digital Content is available in the text

## 1 Introduction

In the past decade, next-generation sequencing and comprehensive genomic profiling have been developed to facilitate detailed classification of cancers, and efficient and unbiased detection of clinically actionable mutations.^[1–3]^ Genetic alterations underlying the cancer pathology reflect cancer biology and might predict response to therapy better than histology.^[4,5]^ It is well established that human epidermal growth factor receptor 2 (*HER2*) is an oncogene, associated with higher risk of recurrence and a poor prognosis in breast cancer. Past studies showed that molecular targeted therapies against HER2 amplification improved prognosis of HER2 positive breast and gastric cancer patients.^[6]^ Gene amplification and overexpression of this protein are seen in approximately 20% to 30% of breast cancer cases.^[7]^ Trastuzumab, the first HER2-directed monoclonal antibody, is a milestone in the treatment of HER2-positive breast cancer at all stages of the disease and in all treatment lines.^[8]^ Four anti-HER2 agents have been currently used for the treatment of breast cancer in Japan. Among these, the combination of trastuzumab with the second HER2 monoclonal antibody pertuzumab has shown substantial effect in *HER2*-amplifed breast cancer.^[9,10]^ The HER2 pathway also plays a key role in pathogenesis of gastric cancer, in which 1.2% to 9% of patients are HER2-positive.^[11]^ This second indication for trastuzumab has been approved in Japan for HER2-positive advanced or recurrent gastric cancer. *HER2* amplification and mutations have emerged as oncogenic drivers and therapeutic targets not limited to breast and gastric cancer, but also in a variety of cancers^[12]^ such as lung,^[13]^ colorectal,^[14]^ bladder,^[15]^ and biliary,^[16]^ urothelial carcinoma,^[17]^ gynecologic,^[18]^ and head and neck cancers.^[19]^ Even if an actionable gene mutation is found in these cancers, the incidence of *HER2* amplification is less than 5%.^[4]^ Despite its considerable therapeutic potential, the evidence is not matured yet to use the treatment in routine clinical practice. In order to address this unmet clinical need, MyPathway study, a phase 2a study that combines multiple basket studies under an adaptable master protocol to evaluate the efficacy of treatments that target molecular alterations in *HER2* (trastuzumab plus pertuzumab), *BRAF* (vemurafenib), the Hedgehog pathway (vismodegib), or *EGFR* (erlotinib) in patients with tumor types outside current labeling for these treatment regimens, is in progress.^[20]^ Interestingly, Hainsworth et al reported that 30 of 114 patients (26%; 95% confidence interval [CI], 19%–35%) with *HER2 *amplification/overexpression had objective responses to treatment with trastuzumab plus pertuzumab.^[20]^ The objective responses were seen in 9 primary tumor types: colorectal, bladder, biliary, salivary gland, non-small cell lung, pancreas, ovary, prostate, and skin cancers.

In consideration of the MyPathway study, we designed a histology-independent phase 2 basket trial of combination therapy with trastuzumab and pertuzumab in Japanese patients with cancer types other than breast and gastric cancers harboring *HER2* amplification.

### 1.1 Objective

The objective of this trial is to evaluate the safety and efficacy of combination therapy with trastuzumab and pertuzumab in patients with locally advanced or metastatic solid cancers harboring *HER2* amplification such as bile duct, urothelial, uterine, ovarian, and other solid cancers.

## 2 Methods

### 2.1 Trial organization and ethical matters

JUPITER trial (A Japanese basket trial using comprehensive genomic profiling informed tumor-agonistic therapy) is an investigator-initiated clinical trial. The study protocol (Ver. 1.0, 2019/1/7) was designed by the study initiators at the Cancer Center, Tokyo Medical and Dental University, Tokyo, Japan, and an additional 6 study sites will participate (Supplemental Digital Content Table S1). The study protocol and the informed consent form were approved by the institutional review board at Tokyo Medical and Dental University (approval number 2018–1005) and at each participating study sites. All participating patients will provide written, informed consent before initiation of any study-specific procedures (Supplemental material). The study is conducted in accordance with the ethical principles originating in or derived from the Declaration of Helsinki and good clinical practice guidelines. The trial was registered in jCRT (Japan Registry of clinical Trials) as jRCT2031180150 (Date of registration February 25, 2019).

### 2.2 Study design and participants

This trial is a Japanese multicenter, single-arm, basket phase 2 study in patients with solid cancers harboring *HER2* amplification that have progressed with standard treatment, or rare cancers for which there is no standard treatment. Target cancers include bile duct, urothelial, uterine, ovarian, and other solid cancers. Breast, gastric and colorectal cancers are excluded from this study because these indications have been approved or clinical trials are ongoing.

**Figure 1:**
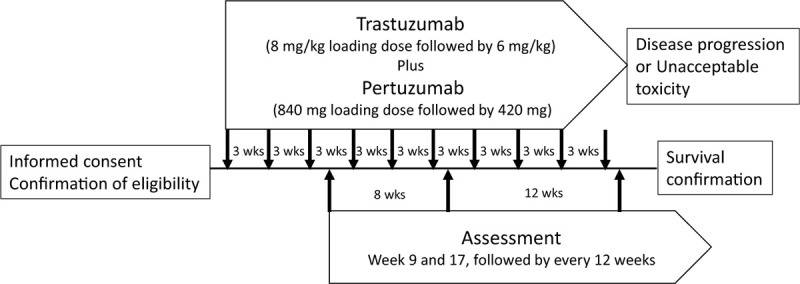
Study design. After informed consent and eligibility confirmation, trastuzumab and pertuzumab are administered every 3 wk until disease progression or unacceptable toxicity. Response assessment is performed at weeks 9 and 17, followed by every 12 wk.

Inclusion and exclusion criteria are shown in Table 1. Patients meeting the eligibility criteria will be accrued in this trial. Patients are treated with intravenous trastuzumab (8* *mg/kg loading dose followed by 6* *mg/kg maintenance dose) and pertuzumab (840* *mg loading dose followed by 420* *mg maintenance dose) every 3 weeks until disease progression, experience of unacceptable adverse events (AEs), death, or withdrawal of informed consent (Fig. 1). No interim analyses are planned in this trial. The trial schedule is shown (Supplemental Digital Content Table S2). The study will be conducted between March 2019 and March 2021, and the data cutoff date will be September 2020. No compensation was provided for participation. In case of adverse events, care is provided without additional financial burden for the participants.

**Table 1:**
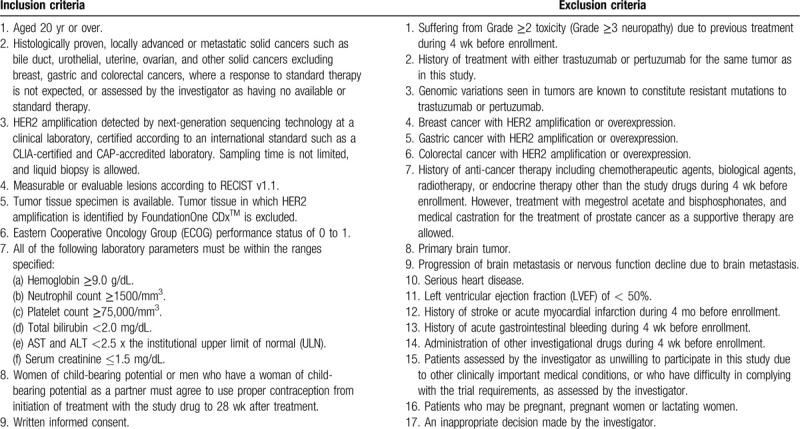
Participant eligibility criteria.

### 2.3 Drug supply

The monoclonal antibodies trastuzumab and pertuzumab are provided by Chugai Pharmaceutical Co., Ltd., Tokyo, Japan, and stored at each study site.

### 2.4 Interventions

Dose and regimen of trastuzumab and pertuzumab are in accordance with the Japanese package inserts for each product.^[21,22]^ The loading dose in the first cycle and the following maintenance dose after cycle 2 of trastuzumab are 8* *mg/kg and 6* *mg/kg, respectively. The loading dose in the first cycle and the following maintenance dose after cycle 2 of pertuzumab are 840* *mg and 420* *mg, respectively. Both drugs are administered intravenously and the administration sequence is not specified. The investigator should confirm that no infusion-related reactions occur during administration and 60* *minutes after administration (30* *minutes observation after cycle 1). The administration cycle will be repeated every 21 days (3 weeks) until disease progression, the patient experiences unacceptable AEs, death, or withdrawal of informed consent. If the previous administration interval is 6 weeks or longer, administration of loading doses of trastuzumab and pertuzumab is re-started, followed by maintenance doses in the following cycles. If an administered dose is <50% of the loading dose due to infusion-related reactions or other events, if possible, the remaining dose is administered within 1 week of starting administration, and the maintenance dose is administered at day 1 of the next cycle (day 22 from the first cycle). If an administered dose is ≥50% to <75% of the loading dose, if possible the remaining dose is administered within 2 weeks of starting administration. If an administered dose is ≥75% of the loading dose, administration of the remaining dose is not necessary. If reduction of left ventricular ejection fraction or AEs for which administration is considered inappropriate are observed, administration is suspended until resolved. Dose reduction is not allowed when re-administration is started. Treatment can be continued after data cutoff according to a separate protocol. Prohibited concomitant medications and therapies are as follows:

(1) other anti-neoplastic agent,
(2) other investigational agent,
(3) radiation, except for palliative radiation for non-targeted lesion,
(4) Immune suppressive agents (high-dose steroid, tumor necrosis factor-alpha inhibitor) (Supplemental Digital Content Fig. S1).

The investigator can provide appropriate supportive care such as antihistamines and/or steroids for the treatment of infusion reactions; antidiarrheal agents; palliative surgery; palliative radiotherapy for bone metastases; and central venous catheterization, preferably 7 days before initiation of treatment. There is no limitation to subsequent treatment after termination of this study, but it should be started after all required examinations are completed.

### 2.5 Efficacy endpoints

The primary endpoint for efficacy is the objective response rate (ORR), the proportion of patients who achieve complete response or partial response. Responses are assessed by independent central radiology imaging review (ICR) according to the Response Evaluation Criteria in Solid Tumors (RECIST) version 1.1. Secondary efficacy endpoints include ORR assessed by the investigator; progression free survival (PFS), defined as the time from the day of initiation of treatment to radiologic progression assessed by ICR or death by any cause; overall survival (OS), defined as the time from the day of initiation of treatment to death from any cause; and duration of response, defined as the time of first documentation of an objective response to the date of tumor progression/death. Exploratory efficacy endpoints are the ratio of PFS in the latest treatment to PFS in this trial, and consistency of accuracy and sensitivity of *HER2* amplification using FoundationOne CDx and other genetic tests.

### 2.6 Efficacy assessment

The first time point for the primary assessment is week 8, and the second analysis is performed in week 16 after initiation of treatment. If duration of treatment is ≥16 weeks, assessment is performed every 12 weeks until treatment termination. Tumor shrinkage and progression are assessed using RECIST version 1.1 by ICR. The investigators evaluate efficacy outcomes by themselves, according to the standardized efficacy assessment method, and submit all image data captured at screening and during treatment to ICR.

### 2.7 Safety assessment

Safety is assessed according to AEs, laboratory tests (hematology and biochemistry), vital signs, body weight, 12-lead electrocardiogram, and echocardiogam. AEs are graded using the common terminology criteria for adverse events v 5.0 (common terminology criteria for adverse events v5.0-JCOG). All AEs are to be documented and the investigator will assess intensity, severity, and relatedness of an AE. All serious AEs is reported according to a standardized serious AEs report form.

### 2.8 Efficacy and safety assessment committee

The independent committee consists of 3 physicians (medical oncologists). They recommend continuation, changing or discontinuation of the trial to the investigators, based on evaluation of safety information and efficacy information, as necessary.

### 2.9 Statistical analysis

#### 2.9.1 Sample size

Assuming that the threshold response rate is 5% and the expected response rate is 20%, the sample size was calculated to be 38 at a one-sided level of significance of 2.5%, with power of 80%. The threshold response rate of 5% was set based on the response rate of trastuzumab which was administered to patients without information about gene mutations, in the phase 1 trial.^[23]^ In the MyPathway study, the response rate to a combination of trastuzumab and pertuzumab was 26% in patients with solid cancers with *HER2* amplification, and 20.8% in those excluding colorectal cancer. Therefore, the expected response rate of 20% was set as a clinically significant response rate.

#### 2.9.2 Statistical analysis plan

Safety analysis will be performed for subjects in the safety analysis set (SAF) and efficacy is analyzed for the full analysis set. The SAF consists of subjects who receive the study drug and have a safety measurement. The full analysis set includes the SAF subjects who have at least 1 efficacy measurement after the first treatment. The cut-off date for the primary analysis is the time when the imaging evaluation at week 3 of the last enrolled patient is completed. The primary efficacy endpoint, ORR will be calculated based on data obtained from RECIST version 1.1 by ICR, and the 95% CI will be estimated using the Clopper–Pearson method. For the secondary efficacy endpoint, ORR based on the data assessed by the investigators and the 95% CI will be calculated. The survival curves for PFS and OS will be estimated using Kaplan–Meier method. The median PFS and OS with their 95% CIs will be calculated. Duration of response will be estimated using the Kaplan–Meier method for the subjects’ response to the treatment, and the median and the 95% CI will then be estimated.

Subgroup analyses will be performed for primary and secondary endpoints for each cancer type. The response rate in each cancer type will also be estimated using the Bayesian information-borrowing approach. Safety will be assessed for all patients who receive at least 1 treatment with the combination of trastuzumab and pertuzumab. All analyses will be conducted with SAS version 9.4 (SAS Institute. Inc., Cary, NA).

### 2.10 Data collection and monitoring

All data are collected via a case report form using an electronic data capture system. Monitoring is conducted independently by a contract research organization according to good clinical practice guidelines. Monitoring will be conducted periodically during the trial to confirm the trial is conducted in accordance with the study protocol. Data will not be publicly available.

### 2.11 Auditing

The contract research organization will perform a planned audit independently from monitoring. Regulatory agencies may also conduct a regulatory inspection of this trial. These audit/inspections can occur at any time during or after completion of the study. The investigator and study site agree to allow the auditor/inspector direct access to all relevant documents. The auditor/inspector can ask the investigator and study site for access to the storage room containing the study products, the pharmacy, and the clinical trial facility.

### 2.12 Dissemination policy

Trial result will be published as a press-release, or in publication. Also, the result will be presented in academic meetings.

## 3 Discussion

JUPITER trial is a Japanese phase 2, multicenter, basket trial to evaluate the efficacy and safety of combination therapy with trastuzumab and pertuzumab in patients with locally advanced or metastatic solid cancers harboring *HER2* amplification.

Quartino et al^[24]^ reported that no evidence of a drug-drug interaction effect was observed between trastuzumab and pertuzumab in the neoadjuvant setting of early breast cancer, and there was no association between the pertuzumab serum concentration and pathological complete response within the dose range of pertuzumab (20–100* *μg/mL), suggesting no dose adjustments is needed for patients with lower exposure. Furthermore, the same doses and regimens used in breast are applied in patients with solid cancers other than breast and gastric cancers in the MyPathway study.^[20]^ The doses and regimens of trastuzumab and pertuzumab approved for use in breast cancer in Japan were used in this study.

The development of trastuzumab and pertuzumab in breast and gastric cancers was conducted using an organ-specific cancer approach in these cancers according to conventional randomized control trials. Instead of focusing on single cancer type, our trial design is a basket study focusing on *HER2* amplification, regardless of the site or origin of the cancer. Efficacy and safety is evaluated in 1 protocol in a variety of cancers harboring *HER*2 amplification such as bile duct, urothelial, uterine, ovarian, and other solid cancers. In our study, we use *HER2* gene amplification defined by next-generation sequencing technology as a biomarker, instead of protein overexpression and fluorescent in situ hybridization because there are reports that selection of patients using genomic biomarkers revealed better clinical outcomes including higher median ORR, and prolonged median PFS and OS than use of a protein biomarker.^[25,26]^ The incidence of *HER2* amplification in our target cancers is reported to be less than 5%.^[4]^ Although considerable therapeutic potential is expected in these rare cancers, a conventional development strategy requires a randomized controlled trial to obtain appropriate indications. Conversely, our basket study can identify a rare cancer that responds to anti-HER2 agents. This is a paradigm shift to genomic biomarker-driven personalized medicine (precision medicine) in cancer treatment, as pembrolizumab has been approved based on the presence of a biomarker rather than the cancer location, without evidence from a randomized controlled trial for each cancer types.^[27]^

Several limitations is considered in relation to our trial. First, molecular diversity is known to exist within tumor sites of individual patients, resulting in underestimation of the genomic complexity of solid cancers.^[28]^ Furthermore, *HER2* amplification appears to be an actionable target in some tumor types, but not all. Liquid biopsy might be extremely helpful in understanding or characterizing specific cancer information in real-time fashion, using a simple blood draw, but still is under development for routine clinical use.^[29]^ Second, the planned number of patients enrolled in this trial is 38, which may be adequate to explore efficacy in *HER2*-amplified solid tumors. However, this number is too small for evaluation of the efficacy in each type of cancer, particularly where no response is seen. Therefore, there is a risk to miss potential efficacy due to the small number of patients.

The results of our trial will advance clinical and scientific knowledge about the treatment of rare, locally advanced *HER2*-amplified solid cancers using the combination of trastuzumab and pertuzumab.

## Acknowledgments

We thank Kazuhiko Arakawa, Jyunko Yokobori, Mika Ohki, and Eriko Takamine for their outstanding contribution to this study as clinical research coordinator and genetic counselor. We appreciate Tomomi Urata, Fumi Komashaku, and Akiko Noguchi for their administrative support. This trial cannot be accomplished without exceptional support from Drs. Yasuhito Yuasa and Kusano (University Research Administration: URA) at Tokyo Medical and Dental University) and Dr. Ryuji Koike at Medical innovation promotion center at Tokyo Medical and Dental University.

## Author contributions

**Akihiro Hirakawa:** study design, statistical analysis plan, reviewing manuscript.

**Eri Ishibashi:** concept generation, protocol development, writing manuscript, reviewing manuscript.

**Hirotoshi Dosaka-Akita, Chikashi Ishioka, Hisahiro Matsubara, Hiroshi Nishihara, Naoko Aragane, Shinichi Toyooka, Manabu Muto:** site PI, accrual of patients, reviewing manuscript.

**Kenta Takahashi:** concept generation, protocol development, accrual of patients, reviewing manuscript.

**Sadakatsu Ikeda:** study design, protocol development, accrual of patients, writing manuscript, reviewing manuscript.

**Satoshi Miyake:** overall supervise, reviewing manuscript.

Toshio Kubo, Yohei Harada, Hideyuki Hayashi, Masayuki Kano, Yasuhi Shimizu, Hidekazu Shirota, Yukiko Mori: site manager, accrual of patients, reviewing manuscript.

**Ukihide Tateishi:** protocol development for imaging review and response criteria, reviewing manuscript.

## Supplementary Material

Supplemental Digital Content

## Supplementary Material

Supplemental Digital Content

## Supplementary Material

Supplemental Digital Content
