# Supplementary material for: A phase 2 basket trial of combination therapy with trastuzumab and pertuzumab in patients with solid cancers harboring human epidermal growth factor receptor 2 amplification (JUPITER trial)
Source: Medicine (Baltimore). 2020 Aug 7;99(32):e21457. doi: 10.1097/MD.0000000000021457 (PMC7592999; doi:10.1097/MD.0000000000021457)
Supplement: Supplemental Digital Content [file medi-99-e21457-s001.docx]

**Supplemental Digital Content**

**Table S1** List of site principal investigators

| Investigator | Institution |
| --- | --- |
| Yasushi Shimizu | Department of Medical Oncology, Faculty of Medicine & Graduate School of Medicine, Hokkaido University, Sapporo, Japan |
| Hisahiro Matsubara | Department of Frontier Surgery, Chiba University, Chiba, Japan |
| Sadakatsu Ikeda | Center for Innovative Cancer Treatment, Tokyo Medical and Dental University, Tokyo, Japan |
| Yasuo Hamamoto | Genomics Unit, Keio Cancer Center, Keio University School of Medicine, Tokyo, Japan |
| Manabu Muto | Department of Therapeutic Oncology, Graduate School of Medicine, Kyoto University, Kyoto, Japan |
| Toshio Kubo | Department of General Thoracic Surgery and Breast and Endocrinological Surgery,, Okayama University Hospital, Okayama, Japan |
| Naoko Aragane | Division of Hematology, Respiratory Medicine and Oncology, Department of Internal Medicine, Faculty of Medicine e, Saga University, Saga, Japan |
| Hidekazu Shirota | Department of Clinical Oncology, Tohoku University Hospital, Sendai, Japan |
