# Supplementary material for: A phase 2 basket trial of combination therapy with trastuzumab and pertuzumab in patients with solid cancers harboring human epidermal growth factor receptor 2 amplification (JUPITER trial)
Source: Medicine (Baltimore). 2020 Aug 7;99(32):e21457. doi: 10.1097/MD.0000000000021457 (PMC7592999; doi:10.1097/MD.0000000000021457)
Supplement: Supplemental Digital Content [file medi-99-e21457-s002.docx]

**Supplemental Digital Content**


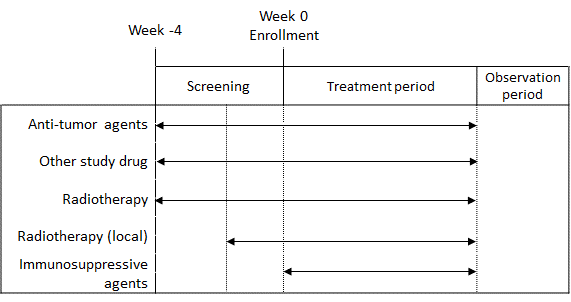


**Fig. S1** Prohibited concomitant medications and treatments, and the period in which they are prohibited.

Immunosuppressive agents include chronic or high doses of oral steroids (> 10 mg/day in terms of prednisolone) and tumor necrosis factor α inhibitors.
