# Supplementary material for: A phase 2 basket trial of combination therapy with trastuzumab and pertuzumab in patients with solid cancers harboring human epidermal growth factor receptor 2 amplification (JUPITER trial)
Source: Medicine (Baltimore). 2020 Aug 7;99(32):e21457. doi: 10.1097/MD.0000000000021457 (PMC7592999; doi:10.1097/MD.0000000000021457)
Supplement: Supplemental Digital Content [file medi-99-e21457-s003.docx]

**Supplemental Digital Content**

**Table S2** Study schedule

| Assessment item | Screening period ^1^ | | Treatment period | | Termination | Observation | Follow-up |
| --- | --- | --- | --- | --- | --- | --- | --- |
|  |  |  | Cycle  Day 1 ^2^ | Assessment of tumor and cardiac function | Termination of treatment | 30 Days after end of treatment | Date of data cutoff |
| Acceptable range (days) | -28 − -1 | -7 − -1 | ±3 | ±7 | ±3 | ±3 | ±14 |
| Informed consent | X ^3^ |  |  |  |  |  |  |
| Enrollment |  | X |  |  |  |  |  |
| Patient characteristics | X |  |  |  |  |  |  |
| History of cancer treatment | X |  |  |  |  |  |  |
| Medical history/complications | X |  |  |  |  |  |  |
| Genetic analysis | X |  |  |  |  |  |  |
| Physical examination | X |  | X |  | X | X |  |
| Vital signs | X |  | X |  | X | X |  |
| Height | X |  |  |  |  |  |  |
| Body weight | X |  | X |  | X | X |  |
| Hematology |  | X | X |  | X | X |  |
| Blood chemistry |  | X | X |  | X | X |  |
| Blood coagulation tests |  | X | X |  | X | X |  |
| Tumor marker |  | X | X ^4^ |  | X |  |  |
| Infectious disease testing | X^5^ |  |  |  |  |  |  |
| Pregnancy test, if needed | X |  |  |  |  |  |  |
| Chest X-ray | X |  |  |  | X |  |  |
| 12-Lead ECG | X |  |  | Every 8 weeks in cycles 1 and 2, followed by every 12 weeks | X |  |  |
| MUGA or echocardiography | X |  |  |  | X |  |  |
| Tumor assessment (CT, MRI) | X |  |  |  | X |  |  |
| Administration |  |  | X |  |  |  |  |
| Adverse events |  |  | X | X | X |  |  |
| Concomitant medication/therapy |  |  | X | X | X |  |  |
| Gene panel test if needed | X^6^ |  |  |  |  |  |  |
| Blood sampling for biomarker identification | X |  |  |  | X |  |  |
| Tissue sampling for biomarker search |  |  |  |  | (X) ^7^ |  |  |
| Follow-up |  |  |  |  |  |  | X |

1. Tumor is detected using CT or MRI.
2. Examination/observation on Day 1 is performed before treatment.
3. Informed consent for exploratory study of biomarkers is taken before starting post-study treatment.
4. Measurement of tumor marker is tested every odd cycle.
5. Screening for infection is performed within the 24 weeks before enrollment.
6. Tumor sample is taken in patients whose *HER2* amplification was detected by means other than FoundationONE CDx after enrollment of the last patient.
7. When disease progression is observed, a tumor sample is obtained in the patient, whose informed consent is obtained before starting post-study treatment.

CT, Computed tomography; ECG, Electrocardiogram; MRI, magnetic resonance imaging; MUGA, multiple-gated acquisition scan
